# The Underutilized Ocular Fundus in Emergency Departments: Current Progress and Future Prospect of Imaging and Artificial Intelligence for Patient Care

**DOI:** 10.1016/j.xops.2026.101338

**Published:** 2026-07-28

**Authors:** Yujie Wang, Wenyi Hu, Xiayin Zhang, Huan Liao, Siegfried K. Wagner, Yueye Wang, Li Li, Ruiye Chen, Xiaomin Zeng, Alex Fan, Myra B. McGuinness, Sam Freeman, Zihan Sun, Anneke Van Der Walt, Gabriel Dawei Yang, Carmel Crock, Can Can Xue, Anran Ran, Gary Ayton, Lucia Le-Kim, Honghua Yu, Tissa Wijeratne, Wei Wang, Yih Chung Tham, Zongyuan Ge, David A. Mackey, Ching-Yu Cheng, Carol Y. Cheung, Franz E. Babl, Hamed Akhlaghi, Mingguang He, Peter van Wijngaarden, Pearse A. Keane, Nancy J. Newman, Valérie Biousse, Tien Yin Wong, Zhuoting Zhu

**Affiliations:** 1 Centre for Eye Research Australia, Royal Victorian Eye and Ear Hospital, Melbourne, Victoria, Australia; 2 Department of Surgery (Ophthalmology), The University of Melbourne, Melbourne, Victoria, Australia; 3 Singapore Eye Research Institute, Singapore National Eye Centre, Singapore, Singapore; 4 Florey Institute of Neuroscience and Mental Health, University of Melbourne, Melbourne, Victoria, Australia; 5 NIHR Biomedical Research Centre, Moorfields Eye Hospital NHS Foundation Trust, London, UK; 6 Institute of Ophthalmology, University College London, London, UK; 7 School of Optometry, The Hong Kong Polytechnic University, Kowloon, Hong Kong, China; 8 School of Psychology, The University of New South Wales Sydney, Kensington, New South Wales, Australia; 9 Guangdong Eye Institute, Department of Ophthalmology, Guangdong Provincial People’s Hospital (Guangdong Academy of Medical Sciences), Southern Medical University, Guangzhou, China; 10 Monash Medical School, Monash University, Melbourne, Victoria, Australia; 11 Centre for Epidemiology and Biostatistics, Melbourne School of Population and Global Health, University of Melbourne, Melbourne, Victoria, Australia; 12 Emergency Department, St Vincent’s Hospital (Melbourne) Limited, Fitzroy, Victoria, Australia; 13 Australian Institute of Health Innovation, Macquarie University, Sydney, New South Wales, Australia; 14 Department of Critical Care, Faculty of Medicine, Dentistry and Health Sciences, University of Melbourne, Melbourne, Victoria, Australia; 15 Department of Neurosciences, Central Clinical School, Alfred Centre, Monash University, Melbourne, Victoria, Australia; 16 Department of Neurology, Alfred Health, Melbourne, Victoria, Australia; 17 Department of Ophthalmology and Visual Sciences, The Chinese University of Hong Kong, Hong Kong SAR, China; 18 Emergency Department, The Royal Victorian Eye and Ear Hospital, East Melbourne, Victoria, Australia; 19 Department of Emergency Medicine, Sunshine Hospital, Western Health, St Albans, Victoria, Australia; 20 Joint Shantou International Eye Center of Shantou University and the Chinese University of Hong Kong, Shantou, Guangdong, China; 21 Department of Neurology, Sunshine Hospital, Western Health, St Albans, Victoria, Australia; 22 Department of Public Health and Psychology, La Trobe University, Bundoora, Victoria, Australia; 23 State Key Laboratory of Ophthalmology, Zhongshan Ophthalmic Center, Sun Yat-Sen University, Guangdong Provincial Key Laboratory of Ophthalmology and Visual Science, Guangdong Provincial Clinical Research Center for Ocular Diseases, Guangzhou, China; 24 Center for Innovation and Precision Eye Health, and Department of Ophthalmology, Yong Loo Lin School of Medicine, National University of Singapore, Singapore, Singapore; 25 AIM for Health Lab, Faculty of Information Technology, Monash University, Melbourne, Victoria, Australia; 26 Airdoc-Monash Research, Monash University, Melbourne, Victoria, Australia; 27 University of Western Australia, Centre for Ophthalmology and Visual Science (Incorporating the Lions Eye Institute), Perth, Western Australia, Australia; 28 School of Medicine, Menzies Research Institute Tasmania, University of Tasmania, Hobart, Tasmania, Australia; 29 Emergency Department, Royal Children's Hospital, Melbourne, Victoria, Australia; 30 The Emergency Research Group, Murdoch Children’s Research Institute, Melbourne, Victoria, Australia; 31 Department of Paediatrics, University of Melbourne, Melbourne, Victoria, Australia; 32 Faculty of Health, Deakin University, Melbourne, Victoria, Australia; 33 Research Centre for SHARP Vision, Hong Kong Polytechnic University, Hong Kong SAR, China; 34 Centre for Eye and Vision Research, Hong Kong SAR, China; 35 Department of Ophthalmology, Emory University School of Medicine, Atlanta, Georgia; 36 Department of Neurology, Emory University School of Medicine, Atlanta, Georgia; 37 Department of Neurological Surgery, Emory University School of Medicine, Atlanta, Georgia; 38 Beijing Visual Science and Translational Eye Research Institute (BERI), Beijing Tsinghua Changgung Hospital Eye Center, School of Clinical Medicine, Tsinghua Medicine, Tsinghua University, Beijing, China

**Keywords:** Ocular fundus examination, Emergency department, Retinal camera, Ocular fundus imaging, Artificial intelligence

## Abstract

**Clinical Relevance:**

The retina is a unique and accessible window to the central nervous system and its vasculature. Ocular fundus examination is crucial for identifying diagnostic red flags indicative of life- and sight-threatening conditions, such as papilledema and central retinal artery occlusion. In the emergency department (ED), prompt recognition of these conditions is essential to reduce the risk of vision loss and serious complications.

**Methods:**

This review summarizes advances in ocular fundus examination, imaging, and artificial intelligence (AI) for emergency care.

**Results:**

The direct ophthalmoscope, a traditional tool for ocular fundus examination by nonophthalmology providers in the ED, is rarely utilized due to technical limitations and lack of user skill and confidence. Recent advancements in retinal imaging technologies have introduced ocular fundus cameras with OCT as valuable alternatives. The advent of nonmydriatic and portable imaging devices has significantly expanded accessibility, enabling remote image interpretation and facilitating teleophthalmology consultations. Moreover, AI, especially deep-learning technology, has demonstrated considerable potential for automated ocular image interpretation. The latest developments in large language models show promise in providing aids in diagnosis and management.

**Conclusions:**

Further research is needed to validate the reliability of AI-powered ocular fundus assessment and to explore how these emerging technologies can be effectively integrated into ED practice to enhance ocular fundus examination and improve patient care.

## Graphical abstract

**Figure:**
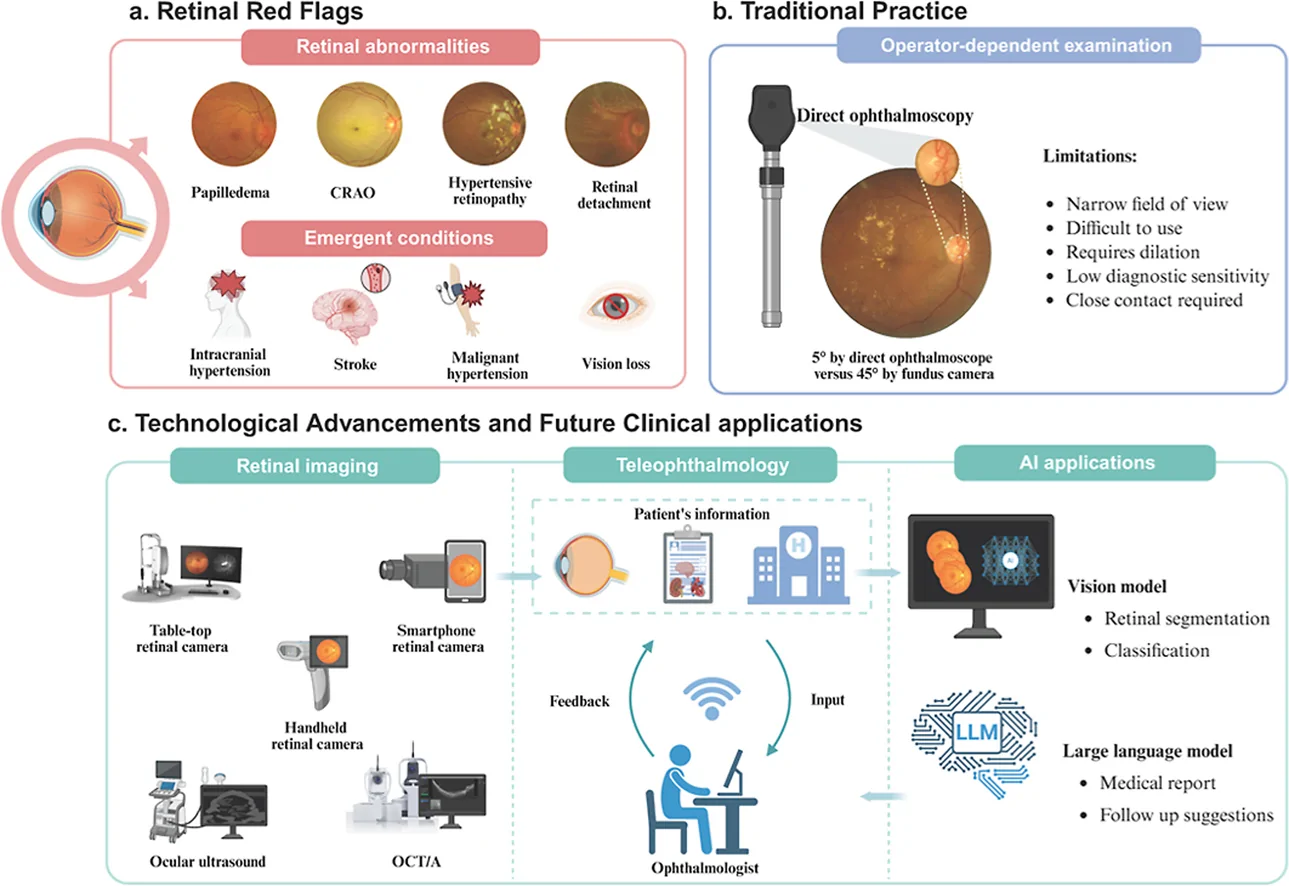

Historically regarded by philosophers as a window to the soul, the eye is now understood by scientists to be intricately linked to overall health.^1^ The retina provides a direct and noninvasive view into the CNS and its vasculature in vivo.^2^ Ocular fundus examination is a convenient, noninvasive, and fast method to directly visualize a part of the CNS and its microvasculature. In the emergency department (ED), it is crucial to identify retinal red flags through examination of the ocular fundus, such as central retinal artery occlusion (CRAO),^3^ papilledema,^4^ and Terson syndrome,^5^ to facilitate timely and accurate diagnosis of vision-threatening and potentially even life-threatening conditions.

Despite its significant clinical relevance, examination of the ocular fundus is infrequently and often inadequately performed by ED providers.^6^^,^^7^ This reflects the technical limitations of direct ophthalmoscopy and the limited proficiency and confidence of most ED providers, driven by insufficient training, minimal practice opportunities, a restricted field of view that reduces diagnostic sensitivity, and the need for patient proximity, which may cause discomfort for both clinicians and patients.

Technological advancements, however, have introduced more accessible and sensitive devices such as easy-to-use ocular fundus cameras, which have generated increasing interest for ocular fundus assessment in EDs. Additionally, artificial intelligence (AI) has advanced rapidly in image recognition, showing strong potential for enhancing ocular fundus examination in EDs.^8^ Artificial intelligence–assisted triage can accelerate diagnosis, optimize resources, prevent vision and even life loss, and enhance both patient experience and staff satisfaction.^9^

This review synthesizes emerging evidence on ocular fundus examination in emergency medicine, highlighting its clinical relevance, advances in ocular fundus imaging technologies, and the growing potential of AI to transform ocular fundus assessment in acute care.

## The Retina as a Neurovascular Window in Emergency Care

### Neurovascular Foundation

The retina represents a specialized extension of the CNS, sharing embryological origin, neuronal architecture, synaptic signaling mechanisms, and immune privilege with the brain.^2^ Retinal ganglion cell axons form the optic nerve, a central white-matter tract, while the blood–retina barrier closely parallels the blood–brain barrier in maintaining neural homeostasis.

The retinal and cerebral circulations are anatomically and functionally homologous. Retinal vessels arise from the internal carotid system and exhibit end-arterial architecture without collateralization, rendering them particularly vulnerable to ischemia.^10^ Both vascular beds demonstrate autoregulatory capacity, high oxygen extraction, and dense capillary networks adapted to high metabolic demand.^11^^,^^12^

**Figure 1:**
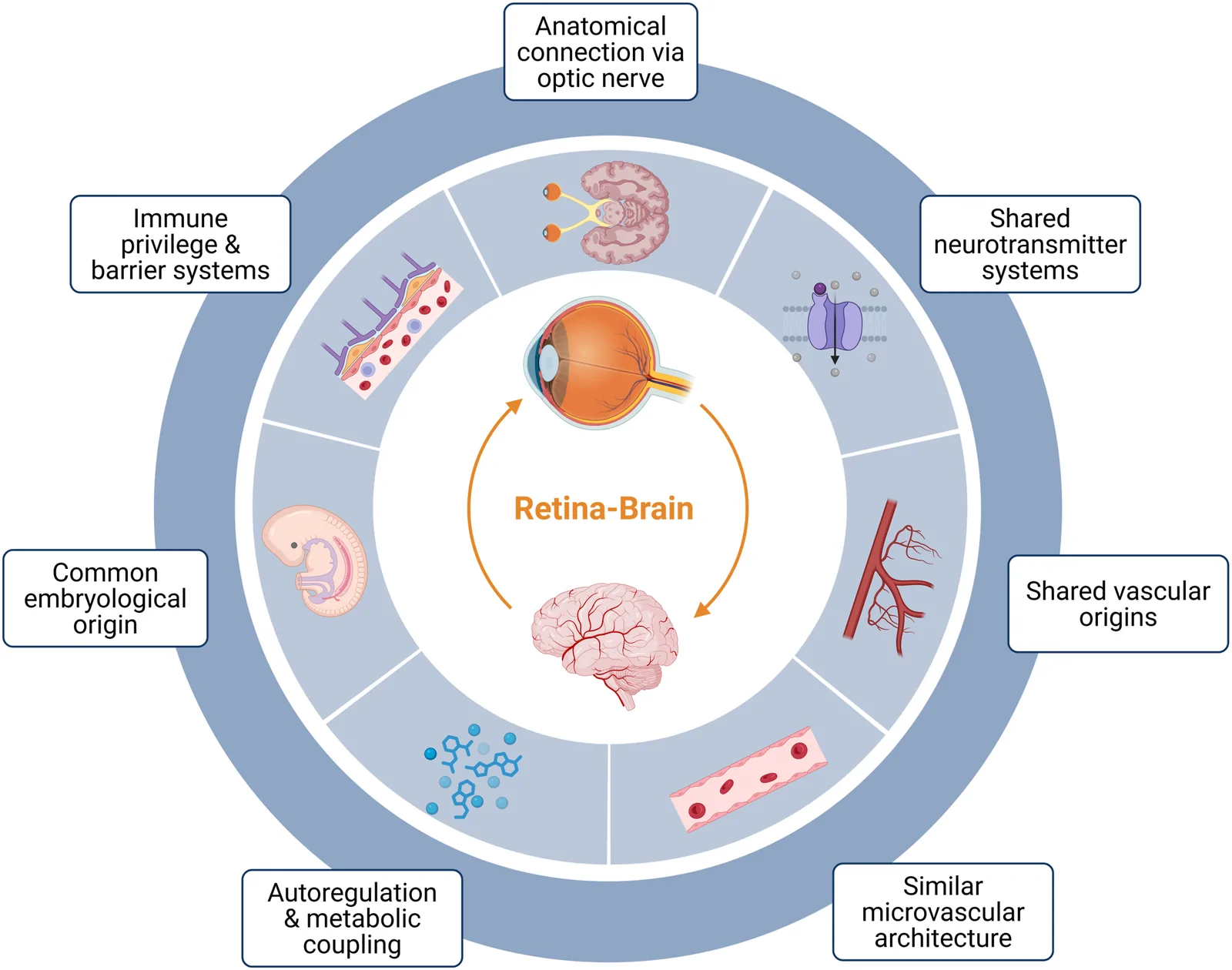
Simplified diagram of the connections between the eye and the brain. This figure was created with BioRender.com.

These shared neurovascular characteristics support the role of retinal imaging as a noninvasive surrogate for detecting cerebral and systemic vascular pathology (Fig 1).

### Ocular Fundus Red Flags in the Emergency Care

**Figure 2:**
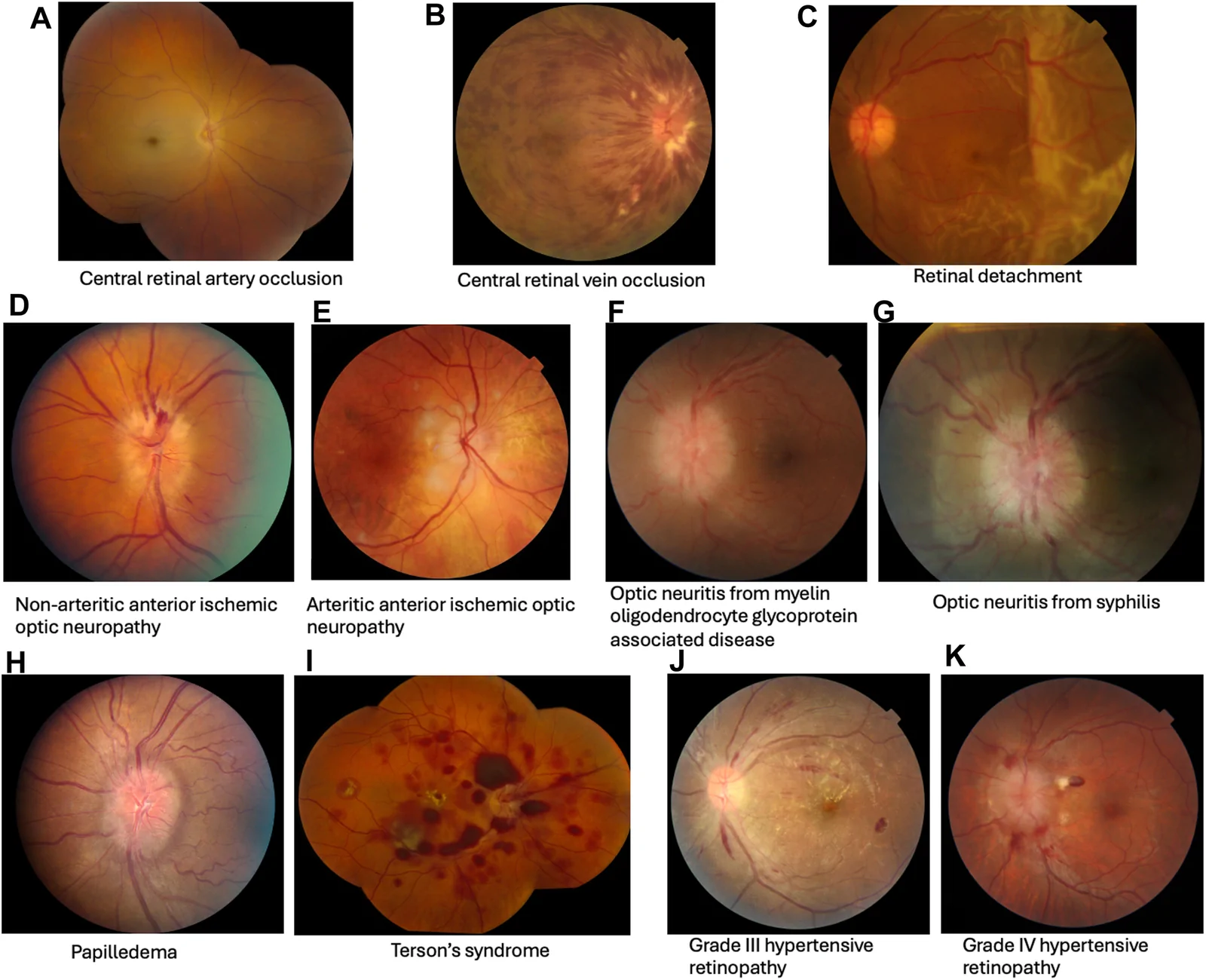
Key ocular red flags indicative of sight- and life-threatening conditions. (**A**) Acute central retinal artery occlusion (courtesy of Valérie Biousse, MD). (**B**) Acute central retinal vein occlusion^13^ (from 1000 Fundus images with 39 categories data set, under the Open Database License and Database Contents License). (**C**) Retinal detachment (courtesy of Honghua Yu, MD, PhD). (**D**) Nonarteritic anterior ischemic optic neuropathy (courtesy of Valérie Biousse, MD). (**E**) Arteritic anterior ischemic optic neuropathy^14^ (from Wilkinson et al 2025, under CC BY-NC 4.0). (**F**) Anterior optic neuritis from myelin oligodendrocyte glycoprotein associated disease (courtesy of Valérie Biousse, MD). (**G**) Anterior optic neuritis from syphilis (courtesy of Valérie Biousse, MD). (**H**) Papilledema (courtesy of Valérie Biousse, MD). (**I**) Terson syndrome from aneurysmal subarachnoid hemorrhage (courtesy of Valérie Biousse, MD). (**J**) Moderate hypertensive retinopathy (courtesy of Honghua Yu, MD, PhD). (**K**) Malignant hypertensive retinopathy (courtesy of Honghua Yu, MD, PhD).

In the ED, ocular fundus findings can signal both sight-threatening ocular disease and life-threatening systemic pathology (Fig 2 and Table 1). Important ocular red flags include retinal whitening with a cherry-red spot or visible emboli in CRAO, diffuse retinal hemorrhages with venous tortuosity and disc swelling in retinal vein occlusion, hyperreflective inner nuclear layer lesions on OCT in paracentral acute middle maculopathy, retinal elevation or corrugation in retinal detachment (RD), and optic disc swelling in anterior ischemic optic neuropathy or optic neuritis. Other urgent abnormalities include infectious retinitis, traumatic retinal injury, and acute macular lesions. Prompt recognition of these findings is essential, as delayed diagnosis may result in irreversible visual loss.

**Table 1:** Summary of Several Key Retinal Red Flags for Life- and Sight-Threatening Conditions

Retinal Red Flags	Presenting Symptoms/Signs	Key Ocular Fundus Findings	Typical Demographic	Critical-Related Conditions
Papilledema	• Headache • Nausea and vomiting • Transient visual obscuration • Diplopia	• Bilaterally swollen, hyperemic discs • Blurring of the optic disc margins	• Depends on cause; idiopathic intracranial hypertension often younger women with obesity	• Brain tumor • Intracranial hemorrhage, • Hydrocephalus • Venous sinus thrombosis • Meningeal process • Idiopathic intracranial hypertension
Terson syndrome	• Sudden vision loss • Blurred vision • Headache • Nausea and vomiting	Preretinal and intraretinal haemorrhages	• Typically adults with acute neurological events	• Subarachnoid hemorrhage • Intracerebral hemorrhage • Traumatic brain injury
Retinal artery occlusion	Sudden, severe, and painless loss of vision	• Cherry-red spot • Retinal whitening • Boxcar segmentation • Attenuated retinal arteries	• Older adults; vascular risk factors	• Atherosclerotic cardiovascular disease risk • Irreversible vision loss • Embolism/thrombus formation • Giant cell arteritis (specially for CRAO)
Retinal vein occlusion	Sudden painless vision loss	• Flame-shaped and dot-blot hemorrhages • Dilated tortuous veins • Cotton wool spots • Optic disc swelling • Macular edema	• Older adults; vascular risk factors	• Atherosclerotic cardiovascular disease risk • Neovascularization
Hypertensive retinopathy stage III/IV	• Blurred vision • Headache	• Flamed-shaped retinal hemorrhages • Cotton-wool spots • Exudates • Arteriovenous nicking • Disc edema	• Severe/uncontrolled hypertension, pregnancy-related hypertension possible	• Malignant hypertension
Retinal detachment	• Sudden onset of floaters • Flashes of light • Shadow/curtain over vision	• Elevated retina • Subretinal fluid • Tears or holes	• Typically middle-aged or older adults; especially those with high myopia, prior ocular surgery, or ocular trauma	• Irreversible vision loss
Anterior ischemic optic neuropathy	• Visual loss • Headache • Scalp tenderness • Pain or fatigue in the jaw muscle	• Pale, swollen optic disc • Cotton-wool spots	• Older adults, especially with giant cell arteritis symptoms	• Giant cell arteritis • Irreversible vision loss • Aortic aneurysm and dissection • Systemic inflammatory response
Optic neuritis	• Blurred vision • Eye pain • Color vision changes	• Normal or swollen optic disc	• Younger adults, more often female	• Irreversible vision loss • Multiple sclerosis and other demyelinating diseases

CRAO = central retinal artery occlusion.

Fundus examination may also reveal clues to systemic emergencies. Key red flags include papilledema in raised intracranial pressure, intraocular hemorrhage in Terson syndrome, retinal vascular and microvascular abnormalities associated with stroke, retinal hemorrhages, exudates, and disc swelling in malignant hypertension, and pale disc edema, cotton wool spots, retinal hemorrhages, or occasionally retinal artery occlusion in giant cell arteritis. These retinal and optic nerve changes may reflect underlying neurological or systemic disease before other clinical signs become evident, highlighting the diagnostic value of fundus assessment in emergency care.

## Current Practice of Ocular Fundus Examination in the ED

### History of the Direct Ophthalmoscope

Owing to its affordability and portability, the direct ophthalmoscope is often the primary fundus examination tool available in most EDs. The invention of the direct ophthalmoscope is attributed to the German physiologist Hermann von Helmholtz in 1851.^15^ He was the first to visualize the human ocular fundus in vivo using a direct ophthalmoscope. Albrecht von Graefe was the first to describe ocular fundus abnormalities, including CRAO and optic disc excavation. Indeed, the advent of the direct ophthalmoscope marked the emergence of ophthalmology as an independent medical specialty.^16^

Direct ophthalmoscopy employs coaxial illumination and viewing beams reflected through a perforated mirror to produce an upright, magnified image of the retina. As a monocular instrument, it does not provide stereopsis.^17^ Because the direct ophthalmoscope’s field of view is only 5 degrees, its high magnification makes this instrument suited for examining the optic disc.^18^ In recent years, the PanOptic ophthalmoscope has emerged as a device offering an expanded field of view of 25 degrees.

### Current Practice of Ocular Fundus Examination in Emergency Departments

Although direct ophthalmoscopes are readily available in EDs, several studies have shown that ED providers do not routinely use this tool.^6^

The initial phase of the Fundus Photography vs. Ophthalmoscopy Trial Outcomes in the Emergency Department (FOTO-ED) study evaluated the use of direct ophthalmoscopy in a US university hospital ED.^19^ The study revealed that only 49 of 350 adult patients (14%) presenting with primary symptoms such as headache, focal neurologic deficit, visual loss, or diastolic blood pressure of ≥120 mmHg underwent ophthalmoscopy. Among these 350 patients, 44 (13%) had relevant ocular findings, including 33 with newly identified ocular fundus abnormalities. Notably, only 5 of these patients were examined by ED providers using ophthalmoscopy, and all were incorrectly assessed as normal.

In a study conducted at 2 large teaching hospitals in the United Kingdom,^20^ 45 of 93 patients (48%) referred to neurology reported not having undergone ocular fundus examination, whereas only 4% could not recall being examined with a stethoscope. A retrospective study at a UK teaching hospital investigated 91 adult patients who presented with sudden severe headaches to the ED or medical admissions over 3 months.^21^ Documentation showed that a complete neurological limb examination was performed on 79 patients (91%) and a cranial nerve examination on 75 patients (86%). However, ocular fundus examination was documented in only 42 patients (48%). An Australian study at a regional hospital ED reported among 120 patients undergoing direct ophthalmoscopy, emergency clinicians correctly identified fundus pathologies in only 1 case.^22^ For patients with normal fundi, 54 out of 91 patients were correctly classified (specificity 59%).

### Barriers to Performing Ocular Fundus Examination in the ED

#### Lack of Skill and Confidence among Clinicians

Direct ophthalmoscopy is notoriously challenging to master, and training in this crucial diagnostic skill is often inadequate. This deficiency can result in a lack of proficiency and confidence among ED doctors in performing direct ophthalmoscopy and identifying ocular red flags.^23^

The prominence of direct ophthalmoscopy in medical education is waning, and it is underrepresented in both training and examinations. This reduced exposure leads to a diminished appreciation of its value among medical students and nonophthalmologist physicians.^24^, ^25^, ^26^ Multiple studies have shown the decline in the percentage of medical institutions that require ophthalmology training across the world, including the United States (16%),^27^ Canada (35.7%),^28^ United Kingdom (65%),^29^ and Australia (63%).^30^ Moxon et al^27^ reported that an estimated 70% of medical graduates are not able to use ophthalmoscope properly in the United States. A 2024 systemic review reported a decline in the length of ophthalmology courses over the last 20 years, with only 26.4% of medical students feeling confident in their ophthalmology knowledge and 34.5% in their clinical skills.^31^ Additionally, surveys conducted in the United Kingdom,^32^ the United States,^33^ and Canada^34^ have shown that most ED providers report feeling undertrained and lacking in confidence in performing and interpreting ocular fundus examinations.

#### Technical Limitations

The direct ophthalmoscope has several significant limitations that contribute to its underutilization in clinical practice.^35^ One of its most notable weaknesses is its narrow field of view. In patients with normal vision, the classic direct ophthalmoscope provides a view of approximately 2 disc diameters, equating to about 7 mm^2^ of the retina. This view becomes even smaller in patients with high myopia. Considering that the average human retina has a surface area of 1204 mm^2^, a clinician would need to examine 172 fields systematically to visualize the entire retina and ensure no significant lesions (up to 2 disc diameters in size) are missed.^36^ This is impractical, even for the most experienced practitioners. Although the PanOptic direct ophthalmoscope provides a wider field of view (25 degrees), it is still not wide enough in most situations. Visibility can also be obstructed by media opacities.^37^ Even when used by ophthalmologists on patients with pharmacologically dilated pupils, direct ophthalmoscopes show limited sensitivity for detecting common conditions such as hypertensive retinopathy.^17^^,^^38^ Pharmacological dilation of the pupils is time-consuming and uncomfortable. Concerns, often overstated, about precipitating angle-closure glaucoma further discourage its use. As a result, direct ophthalmoscopy in the EDs is frequently performed without dilation. This further restricts the field of view and reduces diagnostic sensitivity.^39^ Moreover, the close physical proximity required between the patient and clinician during the use can cause discomfort and may be potentially hazardous, especially in the pandemic era.^40^ The PanOptic ophthalmoscope offers an extended working distance and superior optic disc assessment compared with the direct ophthalmoscope.^41^ The PanOptic Plus enables smartphone-based image capture. However, the lack of Digital Imaging and Communications in Medicine (DICOM) compatibility and secure cloud integration limits compliance with the Health Insurance Portability and Accountability Act, raising concerns about data storage and transfer.

## Technological Advancements in Ocular Fundus Examination

**Figure 3:**
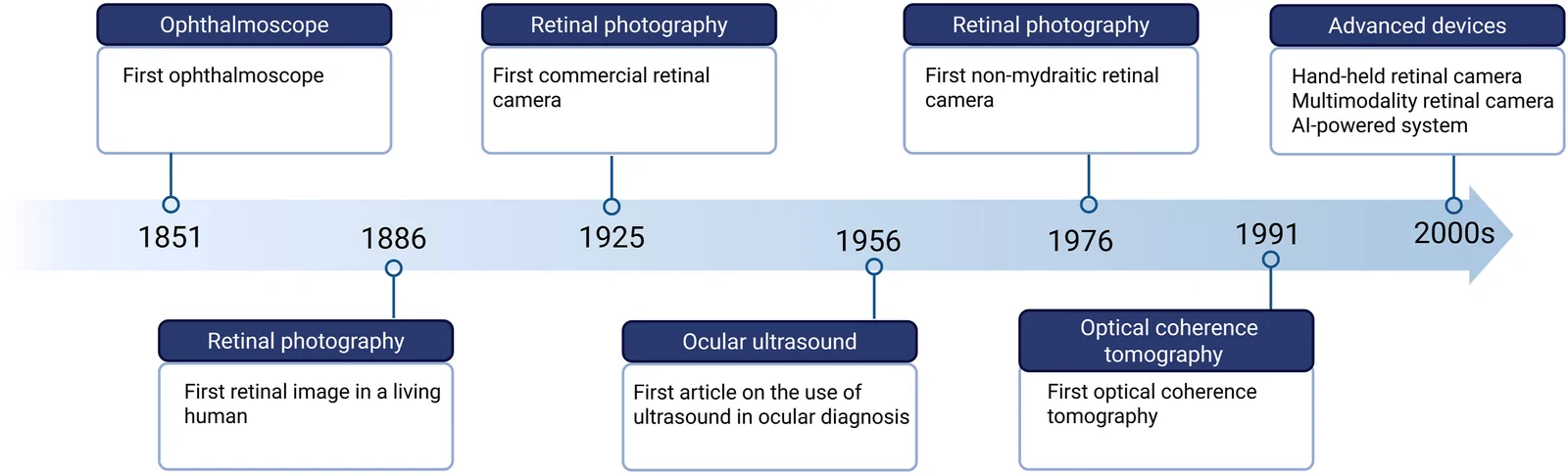
Summary of the development of ocular fundus imaging. This figure was created with BioRender.com. AI = artificial intelligence.

While traditional ocular fundus examination is limited by the shortcomings of direct ophthalmoscopy, advancements in retinal imaging have introduced a variety of modern ophthalmic devices that enhance the quality and accessibility of ocular fundus examination (Fig 3). In the EDs, convenient and noninvasive imaging methods such as nonmydriatic ocular fundus photography and OCT have proven valuable for ophthalmic assessment and support telemedicine by providing high-resolution images for timely and remote diagnosis.

### Ocular Fundus Photography

#### Development of Retinal Cameras

Retinal imaging has undergone substantial evolution since 1886, when Jackman and Webster captured the first retinal photographs in a living human. Key milestones include the first commercial fundus camera by Zeiss in 1925, the introduction of electronic flash and 35 mm film in the 1950s, and the shift to digital imaging after Kodak’s invention of the digital camera in 1975.^42^

Modern ocular fundus cameras now offer stereoscopic imaging and nonmydriatic capabilities using infrared light, enhancing both diagnostic accuracy and patient comfort.^42^, ^43^, ^44^ Compared to direct ophthalmoscopes, these systems provide much broader fields of view and allow secure storage and sharing of images. The integration of automation features, such as autofocus, auto-alignment, and auto-capture, has further improved usability for nonspecialists.^25^ Recent miniaturization of optical systems has enabled the development of compact, portable, and cost-effective devices, expanding access to retinal imaging across primary care, mobile units, underserved regions, and EDs.^44^^,^^45^

#### Nonmydriatic Ocular Imaging

Nonmydriatic fundus photography (NMFP) offers a convenient and efficient method for ocular fundus examination without the need for pupillary dilation. This process can be performed by nonophthalmology providers. Nonmydriatic fundus photography is an excellent alternative to direct ophthalmoscopy, particularly in EDs where the prognostic value of ocular fundus examination has been underappreciated, as well as where limited training and time constraints are common.^46^

Many NMFP come equipped with useful features such as a 45° field of view, autofocus, auto-alignment, user-friendly software, task automation, and image storage. These cameras are easy to understand and operate, allowing health care professionals, including general practitioners and nurses, to conduct examinations after minimal appropriate training.^47^ Captured images can be conveniently uploaded to cloud servers or transferred to storage devices for further analysis. Nonmydriatic fundus photography is increasingly used for screening sight-threatening eye diseases such as diabetic retinopathy (DR). Studies have demonstrated that NMFP provides image quality comparable to mydriatic photography and offers high sensitivity and specificity for diagnosing retinal diseases.^48^^,^^49^

#### Table-Top vs. Handheld Cameras

Traditional table-top ocular fundus cameras are sturdy and capable of producing high-quality retinal images. Most modern systems are DICOM compatible and can be integrated into electronic medical record (EMR) systems. Some advanced models can offer widefield imaging, autofluorescence imaging, and anterior segment visualization. Combined OCT–fundus photography systems are now also available, supporting comprehensive multimodal assessment. Despite the ease of use and the unsurpassed image quality, their higher cost, larger footprint, and the need for stable, upright patient positioning may present practical challenges in ED settings, particularly in busy or resource-constrained environments.

Recent advances in optical miniaturization have led to the development of compact, portable, and more cost-effective alternatives, including handheld and smartphone-based ocular fundus cameras. These portable retinal cameras enable examinations in bedridden or mobility-impaired patients. Several devices have already received regulatory approvals, such as US Food and Drug Administration.^50^ Recent studies have demonstrated that portable fundus cameras can achieve relatively good image quality, and have supported their potentials for reliable diagnostic performance in certain clinical settings.^51^, ^52^, ^53^, ^54^ A systematic review of 11 studies on handheld retinal cameras reported a pooled sensitivity of 83% (95% confidence interval [CI]: 77%–88%) and specificity of 92% (95% CI: 79%–97%) for nonmydriatic images, and a pooled sensitivity of 87% (95% CI: 79%–92%) and specificity of 90% (95% CI: 78%–96%) for mydriatic images.^55^ The study settings were primarily community hospitals, eye clinics or dedicated screening centers. It is important to highlight that most evidence to date has focused on DR, while studies on other fundus pathologies with portable cameras remain limited. A few studies explored the sensitivity and specificity for detecting optic disc edema using the portable fundus devices when compared with the standardized clinical examination results.^53^^,^^56^ Bursztyn et al^53^ reported sensitivities of 71.8%–92.2% and specificities of 81.6%–95.2% using a handheld camera, while specificity fell to 59.2%–92.2% when screening strategies prioritized sensitivity. Similarly, a prospective ED cohort study demonstrated sensitivities of 68.6%–84.3% and specificities of 89.0%–100% for portable fundus photography when images were interpreted by ophthalmologists and emergency physicians.^56^

Of note, portable retinal cameras present certain limitations. Handheld devices are more susceptible to operator handshake and alignment challenges, requiring sustained training to ensure stable, artifact-free imaging.^57^ In addition, most portable models offer a narrow field of view, and their limited resolution may make the detection of subtle retinal changes more challenging.^58^ While some high-end devices perform well, variability in sensitivity and diagnostic reliability remains a concern; for instance, Keenan et al^59^ found marked differences across 3 handheld cameras in detecting DR. Smartphone-based models have low resolution and may require mydriasis for optimal imaging.^60^^,^^61^ Other practical limitations, including fragility, battery dependence, limited storage capacity, and lack of DICOM compatibility in some portable devices, should also be considered when deploying portable retinal cameras in clinical settings.^62^

#### True Color versus Pseudo-Color Retinal Imaging

True-color ocular fundus photography, using white light or trichromatic visible-light illumination, captures the retina in natural hues that closely resemble direct ophthalmoscopic views. Its intuitive visual output facilitates the recognition of ocular pathologies (e.g., extensive hemorrhages, optic disc edema, and macular detachment), making it well-suited for rapid interpretation.

In contrast, pseudo-color imaging (e.g., multicolor or scanning laser ophthalmoscopy) utilizes reflectance at multiple wavelengths (e.g., blue, green, infrared) to construct composite images. Although these do not represent natural colors, they enhance contrast and depth resolution across specific retinal layers. This modality is particularly sensitive to subtle structural changes, such as retinal nerve fiber layer defects, cotton-wool spots, vessel sheathing, and early macular pathology. It often outperforms conventional imaging in detecting microvascular abnormalities,^63^ but makes the optic nerve appearance interpretation very challenging.

#### Fundus Autofluorescence

Fundus autofluorescence imaging noninvasively visualizes lipofuscin accumulation in the retinal pigment epithelium reflecting its metabolic state.^64^ As a functional imaging modality, fundus autofluorescence can detect early disease changes before structural alterations appear on color fundus photography or even OCT. Hyperautofluorescence typically indicates lipofuscin buildup and cellular stress, while hypoautofluorescence suggests retinal pigment epithelium loss or signal masking. Fundus autofluorescence is useful in identifying acute or subclinical conditions such as most maculopathies.

#### Widfield and Ultra-Widefield Imaging

Widefield and ultra-widefield imaging have expanded the assessment of peripheral retinal pathology. Widefield imaging typically captures >50° of the retina, whereas ultra-widefield systems, such as Optos, can acquire up to 200° in a single image, encompassing >80% of the retinal surface.^65^ These systems also allow peripheral visualization through relatively small pupils.^66^ Although ultra-widefield imaging has not been widely studied in the ED, its ability to capture peripheral pathology represents a valuable technological advance, particularly in larger EDs and referral centers where posterior segment emergencies are considered.

#### Ocular Fundus Imaging in the ED

The feasibility and utility of NMFP in the ED has been well-established through several studies. In the initial phase of the FOTO-ED study,^19^ nurse practitioners performed NMFP on patients, achieving diagnostic-quality retinal images in 97% of cases, with an average process time of just 1.9 minutes. Among these images, the quality of the optic disc images was superior to other retinal areas.^67^ In the second phase of the FOTO-ED study, ED physicians reviewed 68% (239/354) of patients’ images using NMFP, a substantial increase compared to the 14% reviewed with direct ophthalmoscopy in the first phase.^68^

The use of NMFP also improved the ability of ED providers to identify relevant retinal abnormalities. Emergency providers identified 16 of 35 relevant findings (sensitivity 46%) and 289 of 319 normal findings (specificity 91%).^19^^,^^68^ Additionally, Thulasi et al analyzed ocular fundus images specifically from 497 patients with headache in the FOTO-ED studies, discovering fundus abnormalities in 42 (8.5%) cases.^69^ Among these patients, 12 had disc edema, nine had optic nerve pallor, six had grade III/IV hypertensive retinopathy, and 15 had isolated retinal hemorrhages. A study conducted in an Australian ED found that NMFP influenced management changes in 52 of 133 enrolled patients.^22^ The diagnostic accuracy for acute fundus pathology improved from 0% to 29% sensitivity and from 59% to 84% specificity when comparing direct ophthalmoscopy to NMFP. A US study showed that implementing NMFP in the ED reduced the patient length of stay for papilledema evaluation and eased the burden on residents and on-call ophthalmologists.^70^ Nonmydriatic fundus photography has proven effective in detecting critical fundus abnormalities, thereby improving the management of ED patients requiring fundus examination and facilitating patient care.^71^ Ocular fundus imaging technologies are increasingly being adopted in EDs, with growing availability supporting more timely diagnosis, triage, and management.^72^

### OCT

#### Development of OCT

OCT is a noninvasive, high-resolution imaging technique that provides cross-sectional images of the retina. Since its introduction in 1991 and commercialization in 1996, OCT has evolved significantly, from early time-domain systems to faster, higher-resolution spectral-domain OCT and deeper-penetrating swept-source OCT.^73^, ^74^, ^75^ The advent of OCT angiography further expanded its utility by enabling dye-free visualization of retinal microvasculature.^76^

While traditional OCT systems are bulky and operator-dependent, recent innovations have introduced compact, portable, and even autonomous devices. Robotic OCT platforms allow contactless imaging without operator assistance, offering particular promise for emergency settings.^77^ Recent studies have also explored handheld, nonmydriatic OCT, which enables flexible use at the bedside.^78^ Additionally, hybrid systems combining OCT and color fundus photography enhance versatility in point-of-care use. These advances are broadening OCT’s accessibility and clinical relevance beyond specialized ophthalmic clinics.

#### OCT’s Clinical Utility in the ED

In ED settings, OCT shows robust potential as a rapid screening tool for critical conditions. Wenzel et al^79^ demonstrated that inner retinal hyperreflectivity observed via OCT could serve as a biomarker for CRAO, with an area under the curve (AUC) of 0.99. Consequently, OCT-based retinal thickness analysis may help identify CRAO patients eligible for reperfusion therapy within a therapeutic window of 4.5 hours, improving timely intervention potential.^80^ Additionally, Chandramouli et al reported OCT’s high diagnostic accuracy for RD in EDs, suggesting its potential as a sensitive screening tool. Emergency department clinicians achieved 97% sensitivity and 93% specificity for RD detection on OCT images, compared to 75% sensitivity and 96% specificity on fundus photographs.^81^ A pilot study by Alilin et al used a robotic OCT system to assess 72 eyes for a broad range of posterior eye conditions such as papilledema, RD, and retinal vascular occlusion. Emergency physicians using OCT achieved 69% sensitivity for any abnormality, 100% sensitivity for urgent conditions, and 64% specificity overall, while providers using standard ophthalmoscopy detected no abnormalities.^82^ Shanmugam et al^83^ assessed 63 eyes from 58 patients in the ED and found that nonmydriatic ocular imaging combining fundus photography and OCT achieved higher diagnostic performance than either modality alone.

### Ocular Ultrasound

The first documented use of diagnostic ocular ultrasound was published by Mundt and Hughes in 1956.^84^ Decades later, in 2002, Blaivas et al conducted one of the earliest studies demonstrating the feasibility of using ocular point-of-care ultrasound (POCUS) in ED settings. Their study showed that emergency physicians could diagnose ocular pathologies in patients presenting to the ED using bedside ultrasound.^85^ Point-of-care ultrasound is a noninvasive, rapid, and easily accessible imaging modality that employs high-frequency ultrasound to visualize intraocular structures, particularly within fluid-filled compartments such as the posterior segment of the eye. In the ED context, ocular POCUS has proven effective for the evaluation of several time-sensitive and vision-threatening conditions, including RD, ocular trauma, CRAO, intraocular foreign bodies, and vitreous hemorrhage.^86^, ^87^, ^88^, ^89^ Point-of-care ultrasound also may allow for indirect assessment of elevated intracranial pressure through measurement of the optic nerve sheath diameter.^90^^,^^91^

However, POCUS is associated with several important limitations that warrant careful consideration. As a real-time, operator-dependent modality, image quality and interpretation are inherently variable, and even experienced ultrasonographers may generate inconsistent findings across different pathologies. Although dedicated ocular ultrasound machines routinely used in eye clinics are very reliable and have high resolution, many of the ultrasound machines used in the ED provide suboptimal imaging of the ocular contents, especially in the hands of ED providers who often do not have the necessary expertise. Without formalized and ongoing training, POCUS carries the risk of fostering overconfidence and misdiagnosis.^92^^,^^93^ Additionally, its relatively low spatial resolution restricts the detection of subtle structural changes or early disease.^94^ The absence of robust, standardized, and secure image storage in many systems compromises reproducibility and hinders reliable use in teleophthalmology compared with static ocular fundus imaging. Furthermore, performing POCUS in certain ocular emergencies, such as suspected globe rupture, may worsen injury.^95^ These constraints suggest that POCUS may be better regarded as a complementary, rather than a substitutive, tool alongside established fundus imaging techniques.

### Teleophthalmology

#### Introduction of Teleophthalmology

Teleophthalmology involves the remote acquisition, transmission, and interpretation of ocular fundus images to support triage, diagnosis, and clinical decision-making, without requiring in-person specialist consultation.^96^ Advances in ocular imaging technologies, such as fundus photography and OCT, along with the development of secure digital platforms, have significantly accelerated its adoption.

The successful deployment of teleophthalmology depends on a robust technological foundation, including standardized data formats, interoperable systems, secure transmission protocols, and appropriate delivery models.^97^ Many modern retinal cameras now support the DICOM standard which encodes both image and patient metadata in a structured format, enabling consistent archiving and transmission. Image transfer and storage must be reliable and secure. Given the wide range of ophthalmic imaging devices used across different ocular modalities, picture archiving and communication system plays a central role in consolidating imaging data and enabling remote access by consulting ophthalmologists. To protect patient data, systems must comply with data security standards such as the Health Insurance Portability and Accountability Act, including the use of encryption, strong passwords, and malware protection. Network bandwidth is also critical, as insufficient upload or download speeds can significantly delay image availability and reduce diagnostic efficiency.^98^

Teleophthalmology is delivered through 2 primary models: store-and-forward (asynchronous) and real-time (synchronous). The asynchronous model, where onsite staff capture images for later review by remote ophthalmologists, is suitable for nonurgent cases and is routinely used for DR screening.^99^^,^^100^ In contrast, the synchronous model is needed for acute conditions seen in the ED, such as CRAO or papilledema, that require immediate input. Synchronous teleophthalmology relies on stable connectivity, streamlined workflows, and readily available on-call ophthalmologists. Interpretation may be handled by a single ophthalmologist for consistency or a shared pool for greater flexibility, with appropriate systems needed to manage workflow and avoid delays.^62^^,^^101^

#### Teleophthalmology in the ED

Technologic developments are catalyzing the implementation of teleophthalmology in ED settings, especially as most EDs lack 24/7 ophthalmology coverage. While some tertiary centers maintain on-call ophthalmologists, a substantial proportion of EDs operate with limited access to eye care professionals, highlighting a critical care gap. In the United States, >50% of rural EDs lacked available eye care professionals.^102^, ^103^, ^104^ Teleophthalmology offers a scalable and potentially cost-effective solution to extend ophthalmic expertise to frontline providers, reduce diagnostic delays, enhance triage, and facilitate timely interventions for emergent ophthalmic presentations. Remote interpretation of ocular imaging studies obtained in the ED can be performed by ophthalmologists who interpret ocular imaging studies on a secure cloud platform or in a patient’s EMR, allowing ED providers to benefit from the results of ocular fundus imaging similar to what happens when a radiologic test is performed on an ED patient. Remote interpretation can be combined with medical recommendations after review of medical records by the interpretating ophthalmologist, such as with teleconsultation, or be associated with a telephone or video live discussion of the patient’s medical condition with the ED provider and even with the patient, such as in teleophthalmology.

Studies have shown that teleophthalmology using nonmydriatic ocular fundus photography and OCT can deliver comparable diagnostic accuracy to in-person dilated eye examinations performed by ophthalmologists.^105^ Teleophthalmology has proven effective in detecting a range of retinal pathologies, including DR, age-related macular degeneration, and RD.^83^^,^^97^^,^^106^^,^^107^

Because nonurgent ocular complaints comprise a large share of eye-related ED visits, integrating retinal imaging with teleophthalmology can streamline triage, minimize unnecessary referrals, and better allocate ED resources to urgent cases. Biousse et al implemented NMFP-OCT imaging in the ED for patients with suspected papilledema, with remote interpretation by ophthalmologists for assessment. This approach reduced ED length of stay by 56% and largely eliminated the need for in-person ophthalmology consultations when papilledema was ruled out remotely.^70^^,^^71^ In addition, it is important to note the overlap between acute vision loss and stroke evaluation, as CRAO is increasingly recognized as an underdiagnosed stroke subtype that may benefit from hyperacute reperfusion therapies and certainly secondary stroke prevention.^108^, ^109^, ^110^ The use of teleophthalmology can assist ED physicians in promptly identifying and managing CRAO, potentially reducing the risk of subsequent stroke and improving patient outcomes.^110^, ^111^, ^112^ Teleophthalmology also offers educational benefits for trainees, enhancing their diagnostic confidence while reducing the burden on both trainees and on-call ophthalmologists.^71^^,^^113^

## The Application of AI to Ocular Imaging in the ED

Artificial intelligence has catalyzed major advances in medical diagnostics by enabling algorithms to extract complex patterns from clinical data.^114^ Artificial intelligence simulates human intelligence through machines designed to perform tasks. Within AI, machine learning develops algorithms that learn from data, while deep learning (DL), a subset of machine learning, uses multilayered neural networks to model complex patterns.^115^ In ophthalmology, DL based vision models have demonstrated expert-level performance in detecting eye diseases.^116^, ^117^, ^118^

The release of ChatGPT in November 2022 marked the beginning of an era of mainstream popularity of large language models (LLMs). Large language models are transformer-based DL architectures trained on large text corpora for advanced language understanding and generation.^119^ More recently, multimodal foundation models, including domain-specific systems such as MedGemma, have demonstrated the capability to integrate both textual and visual data, enabling interpretation of medical images alongside clinical context.^120^^,^^121^ These models support more context-aware clinical decision-making, including referral, investigation, and management.^122^

The convergence of vision models and LLMs holds promise for emergency settings, where AI could support rapid, accurate interpretation of retinal images, facilitate documentation, and enhance clinical decision-making.^123^ However, most evidence remains derived from retrospective studies, and clinical applicability in ED settings is still evolving.

### Vision Models in Ocular Fundus Image Analysis

Deep learning has been widely applied to ocular fundus image analysis across a range of tasks and model architectures. Deep learning approaches in ophthalmology span classification, segmentation, detection, and generative modeling.^124^^,^^125^ Convolutional neural networks including architectures such as ResNet, Visual Geometry Group (VGG) networks, and EfficientNet, have been the most widely adopted, demonstrating strong performance in disease classification and grading tasks such as DR severity grading and glaucoma detection.^116^ For pixel-level analysis, encoder-decoder architectures, most notably U-Net and its variants, have been extensively applied to retinal vessel segmentation, optic disc and cup delineation, and lesion segmentation.^126^ Object detection models, particularly the You Only Look Once (YOLO) family, have also gained traction in ocular diagnostics.^127^ Vision transformers and hybrid convolutional neural network–transformer architectures have more recently shown competitive or superior performance by capturing long-range spatial dependencies, while generative adversarial networks have been explored for data augmentation, image quality enhancement, and cross-modal synthesis.^128^ More broadly, the field has shifted toward self-supervised learning and foundation model approaches.^129^

In emergency care, these advances may support rapid screening and triage, with classification aiding detection of urgent conditions and detection models enabling localization of critical findings. Although largely validated in controlled settings, they highlight potential to assist nonspecialist clinicians in time-sensitive environments such as the ED.

#### Identification of Critical Retinal and Optic Nerve Abnormalities

##### Optic Neuropathies

In recent years, several advanced DL methods have emerged to identify optic neuropathies, including papilledema, optic disc edema specifically from elevated intracranial pressure.^130^ A notable example is the model introduced by the Brain and Optic Nerve Study with Artificial Intelligence (BONSAI) group,^131^ trained on 15 846 mydriatic ocular fundus images from diverse ethnic populations. In an external test of 1505 images, this model achieved an AUC of 0.96, with a sensitivity of 96.4% and a specificity of 84.7%. Further validation was conducted using clinical applicability nonmydriatic ocular fundus data from the FOTO-ED studies,^132^ in which the algorithm successfully distinguished papilledema from normal and other conditions with an AUC of 0.97, a sensitivity of 84.0%, and a specificity of 98.9%. The BONSAI group further tested the algorithm on mydriatic fundus photographs from a multiethnic pediatric population, achieving an overall accuracy of 89.6%.^133^ This underscores the algorithm’s effectiveness in pediatric populations and broad applicability. In addition, this study group also showed the algorithm required significantly less time (25 seconds) than neuro-ophthalmologist image interpretation and performed on par with expert neuro-ophthalmologists.^134^ Building on this model, the BONSAI group further proposed a DL model to classify papilledema severity^135^ that demonstrated impressive performance with an AUC of 0.93, along with accuracy, sensitivity, and specificity of 87.9%, 91.8%, and 86.2%, respectively. The BONSAI group developed another DL model that was successful in distinguishing between optic disc drusen and papilledema.^136^ Other researchers also have reported the value of DL in detecting papilledema from fundus images,^137^ and differentiating pseudo papilledema from true papilledema.^138^ The BONSAI group recently developed and validated a DL model using a data set of 961 color ocular fundus images from 802 patients with anterior ischemic optic neuropathies. This model achieved an AUC of 0.97 and an accuracy of 92.6% in distinguishing arteritic anterior ischemic optic neuropathy from nonarteritic anterior ischemic optic neuropathy in external validation.^139^

##### Retinal Vascular Occlusion

Deep learning technologies have shown remarkable proficiency in detecting retinal vascular occlusion. Ren et al^140^ developed a model to identify retinal vein occlusion from color ocular fundus images, achieving robust performance with an AUC of 0.81, accuracy of 0.78, specificity of 0.65, and sensitivity of 0.91 in the external data sets. The DeepDrRVO^141^ system achieved mean sensitivity, specificity, and F1 scores of 0.93, 0.99, and 0.95, respectively, with a mean AUC of 0.99 to detect retinal vascular occlusion. A multitasking AI system called Ai-Doctor demonstrated exceptional performance in diagnosing retinal vein occlusion (AUC range: 0.98 to 0.99).^142^ Gungor et al^143^ developed a DL model to accurately detect hyperacute CRAO on retinal fundus photographs within the critical 4.5-hour treatment window (AUC: 0.96, sensitivity: 0.93, specificity: 0.85).

##### Hypertensive Retinopathy

Arsalan et al^144^^,^^145^ proposed several DL-based segmentation architectures to aid diagnosis of hypertensive retinopathy. These models were successfully validated on 3 publicly available data sets. Qaisar Abbas et al^146^ developed the DenseHyper system using 4270 retinal fundus images, which achieved sensitivity of 93%, specificity of 95%, accuracy of 95%, and an AUC of 0.96 to detect hypertensive retinopathy. Building on this, the team furtherly developed the hypertensive retinopathy (HYPER-RETINO) grading system to detect related lesions and classify stages.^147^ The system achieved sensitivity of 90.5%, specificity of 91.5%, accuracy of 92.6%, and an AUC of 0.915. Several DL algorithms have also demonstrated good performance in detecting hypertensive retinopathy.^148^^,^^149^

##### Other Emergent Abnormalities

Promising results have been shown for the detection of other emergent retinal abnormalities via DL technologies. For instance, Hideharu et al^150^ developed a DL model achieved a sensitivity of 97.6%, specificity of 96.5%, and an AUC of 0.988 in rhegmatogenous RD detection. Li et al^151^ introduced a cascaded DL system for automated RD detection and macula-on/off status discernment, achieving results comparable to those of experienced ophthalmologists. In a recent study, Ailin et al introduced a DL model to classify OCT images as either referable or nonreferable for ophthalmic consultation. External testing showed an AUC of 0.91, a sensitivity of 95%, and a specificity of 76%, which were comparable to 2 human experts.^152^ In addition, AI can be embedded in fundus cameras to assist in identifying abnormal findings of the retina and optic nerve, generating corresponding heat maps to support emergency physicians in their assessment (Valérie Biousse, MD. Personal communication).

Collectively, these studies demonstrate the potential of DL-based models to detect key ocular emergencies; however, evidence is largely retrospective, with limited prospective validation in ED settings. Cross-study comparisons remain challenging due to heterogeneity in data sets, study design, and outcome definitions.

#### Oculomics and Atherosclerotic Cardiovascular Disease Risk Assessment

Oculomics, a rapidly evolving field, leverages ocular fundus imaging to reveal systemic health insights, enabling prediction of cardiovascular, neurological, and other medical conditions.^153^ Advances in AI now allow automated extraction of relevant biomarkers, offering a noninvasive tool to support triage and risk stratification.^1^^,^^154^

Artificial intelligence–based retinal imaging analysis has demonstrated significant potential in stroke detection and risk prediction. Artificial intelligence–powered retinal features derived from retinal images^155^, ^156^, ^157^ and OCT/A^158^ have been used to identify stroke or assess stroke risk. A landmark study by Jiang et al introduced a DL algorithm for predicting silent brain infarction determined by brain magnetic resonance imaging scans using fundus photographs. The model using retinal imaging alone achieved an AUC of 0.901 for incident event prediction, and an AUC of 0.769 for recurrent stroke prediction.^159^ Additionally, Xiong et al^160^ developed a DL model to detect ischemic strokes and classify them into lacunar and nonlacunar subtypes using OCT angiography images, achieving AUCs of 0.822 for stroke detection and 0.766 for subtype classification in external validation.

Beyond stroke-specific applications, retinal imaging has shown promise in broader cardiovascular risk assessment. Deep learning algorithms have been used to predict blood pressure,^161^ hypertension status,^162^ myocardial infarction,^163^ multiple cardiovascular risk factors, and future cardiovascular events directly from retinal images.^164^, ^165^, ^166^ Besides, retinal imaging combined with AI may also provide insights into systemic causes of acute vision loss, such as giant cell arteritis, with diagnostic performance improving when integrated with multimodal imaging.^167^

In the ED, where patients often present with nonspecific symptoms such as headache or visual disturbance, oculomic approaches may enable opportunistic cardiovascular risk assessment alongside ocular evaluation, potentially supporting more comprehensive triage and earlier intervention. However, most evidence is derived from research settings and population-level data sets, and applicability to individual patient-level decision-making in the ED remains uncertain. Further validation is required before integration into emergency care.

### The Potentials of LLMs

The emergence of multimodal LLMs enables integration of ocular fundus images with patient history to support ocular diagnosis and improve performance across retinal diseases.^168^^,^^169^ Their natural language output also aids communication and assists in report writing.^170^ Christoph et al^171^ benchmarked several open-source vision language models against GPT-4o, showing that open-source models achieved limited diagnostic accuracy (up to 40.4%) in emergency and critical care settings compared with GPT-4o (68.1%). Given patient privacy concerns in health care, open-source LLMs are often preferred within the medical community. This underscores the need for specialized training and optimization to enhance the utility of open-source LLMs in supporting emergency care.

The time-sensitive nature of ophthalmic emergencies, in which delayed intervention risks permanent vision loss, necessitates optimized triage throughout the care continuum. In the pre-ED phase, studies indicate 37%–50.4% of patients presenting with ocular complaints do not require emergency care,^172^^,^^173^ suggesting LLMs could provide previsit triage via telehealth interfaces, reduce unnecessary ED visits through appropriate redirection, and enable early identification of true emergencies requiring urgent care. Within the ED phase, in which patient-flow bottlenecks significantly impact care quality, LLM integration may streamline triage processes to reduce wait times, optimize resource allocation based on acuity levels, and enhance documentation efficiency through automated note generation, potentially yielding measurable benefits, including reduced length of stay, faster discharge processes, improved throughput capacity, and enhanced patient satisfaction scores.^174^, ^175^, ^176^, ^177^ For the post-ED phase, LLMs could facilitate care transitions across various pathways by ensuring appropriate specialty handoffs during acute/subacute inpatient transfers, supporting remote monitoring protocols in hospital-at-home programs, generating personalized aftercare instructions for discharge planning, and facilitating continuity of care in residential care coordination.^178^ As such, the role of LLMs in ED settings remains exploratory and requires further prospective validation.

## Challenges and Future Directions

### Challenges and Barriers

#### Organizational and Systemic Barriers

Promoting ocular fundus examination in the ED requires substantial initial investment, particularly for imaging equipment, with remote interpretation and teleophthalmology introducing additional costs related to digital infrastructure and clinical integration. These financial barriers are especially pronounced in resource-limited settings and have been consistently identified as major obstacles to adoption of these technologies, as demonstrated by several studies.^103^^,^^179^^,^^180^ Although traditional ocular fundus cameras generally exceed the cost of standard ED equipment such as electrocardiogram machines, the development of compact, lower-cost models may enhance feasibility.^181^ Demonstrating the clinical value, such as early detection of emergency conditions, streamlined triage, improved patient outcomes, and prevention of costly diagnostic errors, should be used to help justify institutional or policy-level investment. Additionally, integration of ocular imaging studies into patients’ EMR associated with remote interpretation of ocular imaging studies by ophthalmologists allows for automated charges for these interpretations (similar to what is routinely done in eye clinics), which can cover the cost of a camera very quickly.

Additionally, several technical barriers can hinder effective integration. Retinal image acquisition often requires patient cooperation, which can be difficult in older, immobile, or critically ill individuals, frequently leading to suboptimal image quality. Some imaging systems lack DICOM compatibility or robust application programming interfaces for integration, impeding seamless linkage with EMRs and hindering timely clinical access and longitudinal patient documentation. Infrastructure deficits (e.g., unstable power, limited Wi-Fi, and low-bandwidth internet, especially in rural settings) further constrain teleophthalmology use. In addition, many imaging systems require regular calibration and technical oversight, yet access to trained personnel for maintenance and troubleshooting may be limited in the ED.^62^^,^^101^

Successful implementation also relies on systemic enablers. At the system level, scalability is constrained by the absence of consistent reimbursement structures. In many jurisdictions, ED-based synchronous or asynchronous teleophthalmology consultations are not formally recognized within existing billing models, and insurance coverage varies, thereby limiting institutional incentives for adoption.^182^ However, billing for interpretation of ocular imaging studies should not be an issue in most settings, providing that all necessary steps of image review and documentation of interpretation are followed, similar to what is done by radiology departments. Accessibility is further constrained in underresourced or rural settings, where infrastructure, staffing, and specialist support may be insufficient.^98^ Without coordinated investment and supportive policies, the widespread and equitable implementation of ED-based retinal imaging remains challenging.

#### Human and Operational Barriers

Integrating retinal imaging and teleophthalmology into existing ED workflows requires well-defined clinical pathways and decision support. However, the absence of universally accepted guidelines for ocular fundus photography indications contributes to inconsistent use and underutilization. A recent survey conducted among Australian ED doctors revealed poor interrater agreement on clinical scenarios warranting ocular fundus examination (Wang et al, unpublished data). Additionally, ambiguity surrounding the roles of personnel, specifically for those who should initiate and perform imaging (e.g., ED physicians, nurses, or technicians) and who should interpret and act on the findings (ED clinicians or ophthalmologists), may further complicate implementation.

Most importantly, clinician engagement is essential for adoption.^62^ Limited training, uncertainty in image interpretation, and the perception that ocular assessment falls outside the traditional scope of ED practice may contribute to low uptake.^180^ In some settings, a culture of risk aversion further discourages clinicians from initiating retinal imaging, due to concerns that documented images may expose diagnostic errors or omissions.^72^ Additionally, in high-acuity and resource-constrained ED environments, ocular imaging may be perceived as an added burden, with competing clinical priorities and workload pressures reducing willingness to adopt new tools. It has been suggested that one of the best solutions to these human barriers is increasing ED provider awareness that implementation of ocular imaging in the ED results in the identification and prevention of diagnostic errors such as missed papilledema.^62^ Indeed, the consequences of missing papilledema in a headache patient presenting to an ED where examination of the ocular fundus is not systematically performed are far worse than the risk of misinterpretation of ocular imaging obtained in the ED.^62^^,^^183^

#### AI-Assisted System Barriers

In addition to the previously discussed barriers, the adoption of AI introduces a distinct set of challenges. Addressing these domains is critical for safe and effective implementation.

Artificial intelligence performance is often compromised by imbalanced training data across demographic and clinical variables, leading to diagnostic bias and reduced accuracy in underrepresented populations.^184^ Most large-scale clinical databases are sourced from Asia, North America, and Europe, with limited representation from low- and middle-income countries, contributing to AI's underperformance in these regions.^185^ In addition, rare but critical ocular emergencies such as CRAO and papilledema are particularly challenging, as their low prevalence makes it difficult to obtain sufficient cases for robust model validation. Crucially, the effectiveness of these AI applications in both prehospital and inhospital emergency settings still requires robust evidence-based validation, particularly regarding their impact on improving ED patient flow metrics and enhancing satisfaction levels among both health care providers and patients. These evidence gaps highlight the pressing need for multicenter clinical trials and clinical applicability implementation studies to establish the clinical utility and operational benefits of AI systems across diverse health care settings and patient populations.

Data privacy and governance also represent important considerations in the development and deployment of AI systems. Although anonymization and deidentification are key strategies to protect data privacy, the risk of reidentification remains, necessitating stronger safeguards throughout the AI development and implementation process.^186^

The inherent “black box” nature of AI models complicates understanding, potentially eroding trust in AI applications.^187^ A lack of explainability may reduce clinicians’ willingness to incorporate AI into their diagnostic workflow, especially when the rationale behind the algorithm's recommendation is unclear. Transparency is also important from the patient perspective.^171^ Limited awareness or understanding of AI may pose challenges for informed consent and could affect patients’ comfort with its use, particularly in emergency settings, where individuals may be unwell and less able to participate fully in decision-making. Recent work has highlighted the potential role of explainable AI techniques in improving interpretability such as saliency mapping and feature attribution that provide insight into model decision-making.^125^ However, current approaches remain limited by issues of stability, lack of standardization, and uncertain clinical utility, highlighting the need for further validation before routine clinical integration.^125^

Regulatory and legal uncertainty also pose a substantial barrier to the integration of AI-assisted diagnostic systems in the ED. Compared to AI systems approved for DR screening (i.e., tools used in low-risk and well-defined outpatient settings), AI deployment in emergency care is more complex.^188^ Emergency applications involve high-stakes, time-sensitive decisions across diverse conditions, where diagnostic errors can have severe consequences. While regulatory bodies in regions such as the United States, Europe, and Australia have begun to develop frameworks for AI as a medical device,^189^^,^^190^ current policies are better suited to static algorithms. Adaptive, continuously learning systems remain difficult to regulate. Furthermore, most legal frameworks assign final responsibility to clinicians for AI-assisted decisions, reinforcing the primacy of clinical judgment. While this approach supports patient safety, it may also lead to concerns that increased reliance on AI could introduce additional medico-legal considerations for physicians.

Effective integration into existing ED workflows remains a critical consideration for AI implementation. Prior studies noted that limited interoperability with EMR systems may compel clinical staff to manually transfer AI-generated reports, increasing workload and compromising efficiency.^191^^,^^192^ Furthermore, ensuring intuitive and user-friendly user experience and user interface design is essential, as poorly optimized interfaces developed without end-user input can substantially impede adoption.^193^ However, unlike existing clinically approved autonomous AI systems that are mostly designed to screen for DR,^194^^,^^195^ any AI associated with ocular imaging obtained in the ED should provide a probability of normal versus abnormal, such as the Eyer2 (Phelcom)^196^ or a probability of a specific diagnosis, such as with the nonpublicly available BONSAI deep learning system.^131^^,^^133^ Such algorithms can be used as diagnostic aids in the ED, allowing ED providers to incorporate a probability of the ocular finding into their decision-making process.^132^ This diagnostic aid provided by AI, if included in the picture archiving and communication system, would be no different from what is currently provided by most electrocardiogram machines which deliver a tentative interpretation of electrocardiograms routinely performed in the ED and universally accepted by ED providers.^197^

### Future Directions

#### Technical Advancements

Looking ahead, future tabletop ocular fundus imaging systems may benefit from more integrated functionality and improved usability, such as fully automated operation, hybrid platforms combining true-color fundus photography with OCT, and compatibility with AI-assisted image analysis. Simultaneously, handheld and smartphone-based retinal cameras offer critical advantages (e.g., portability, low cost, and adaptability in remote or resource-limited settings) but face pressing technical challenges. Future efforts should focus on enhancing image quality, enlarging the field of view, improving standardization across devices, and ensuring diagnostic-grade consistency, particularly in nonmydriatic conditions. Critically, all the cameras need to support interoperability standards like DICOM/picture archiving and communication system and EMR interfaces, facilitating secure image transfer and remote diagnostic support. In addition, integration of embedded AI and edge computing could enable real-time image analysis, active image acquisition, and improved operator independence. These ocular fundus imaging systems could demonstrate dual-phase utility: in the pre-ED phase, they enable precise triage and intervention through telehealth platforms, facilitating home-based assessments and appropriate care pathway recommendations; in the post-ED phase, they support transitional care by enhancing monitoring capabilities during patient transfers from ED/inpatient settings to home or alternative care facilities.

Complementing these hardware innovations, telemedicine integration through remote monitoring and virtual observation units has demonstrated capacity to accelerate safe ED discharges, reduce inpatient bed occupancy, and maintain care continuity in home hospital programs.^198^^,^^199^ However, rigorous multicenter trials remain imperative to quantify the clinical outcomes, operational efficiencies, and cost-benefit ratios of these technologies across diverse health care ecosystems. Priority research domains should include validation of diagnostic concordance between portable and conventional systems, and assessment of their longitudinal impacts on patient flow metrics and satisfaction indices.

#### Prospective Clinical Trials

Several prospective studies have demonstrated the feasibility and utility of ocular imaging and tele-ophthalmology in ED settings, showing improvements in clinical decision-making, ED workflow efficiency, and support for remote consultations.^56^^,^^70^^,^^83^^,^^111^^,^^112^^,^^200^^,^^201^ For instance, the Emory University team integrated hybrid nonmydriatic fundus cameras and OCT into ED workflows.^62^ Their study demonstrated consistent use of the imaging system by ED physicians and implementation feasibility over a 1-year period. These applications have demonstrated clinical value in facilitating the rapid diagnosis or exclusion of ophthalmic emergencies, including papilledema,^70^^,^^202^ CRAO,^112^ and RD.^83^ The New York University team validated the same hybrid camera in the ED, showing it can provide high-quality images for rapid posterior segment diagnosis.^203^ More prospective clinical trials are needed to verify the findings across different regions/countries, where ED environments, workflows and health care systems will be diverse. The deployment of fundus imaging and teleophthalmology must account for heterogeneity across emergency care settings. Some EDs, particularly in well-resourced urban centers such as Singapore, are integrated with specialist eye hospitals and benefit from on-call ophthalmologists.^204^ In contrast, many EDs in rural or underserved regions (e.g. remote United States, remote Australia, China, and India) lack routine ophthalmic support.^205^, ^206^, ^207^ These disparities underscore the need for context-specific models—such as portable imaging devices, AI-enabled diagnosis, and asynchronous tele-ophthalmology—to bridge diagnostic gaps and ensure equitable access to care.^208^

In addition, as previously noted, ocular fundus imaging has the potential to identify a broader range of sight-threatening and systemic neurovascular conditions. A retrospective study across 21 EDs in Kaiser Permanente Northern California found that, among patients presenting with visual complaints, use of ocular fundus cameras was associated with shorter ED length of stay, higher 72-hour eye clinic follow-up rates, and more definitive diagnoses.^209^ Emerging studies are exploring the use of ocular fundus imaging on patients with vision or neurologic complaints in ED.^210^^,^^211^ Future prospective studies are needed to assess ocular imaging’s role as an ED triage tool capable of detecting diverse presentations, improving referral accuracy and streamlining care pathways.^210^ Additionally, the effectiveness of ocular fundus imaging in improving patient outcomes must be documented in future studies.

There is strong potential for the ocular fundus examination in EDs to generate downstream cost savings and optimize resource use. To support broader adoption and inform health policy, robust cost-effectiveness analyses are essential. Prospective clinical trials can provide critical empirical data for health economic evaluations, such as user uptake, clinical applicability model performance, and costs of staffing, training and resource use.^212^ Demonstrating cost-effectiveness would help justify investment from hospitals and health systems, and support policy-level decisions for widespread integration of ocular imaging into ED care pathways. Demonstration that the presence of ocular fundus cameras in EDs prevents devastating and costly diagnostic errors is an important step. Missed papilledema commonly results in poor patient outcomes, sometimes with medical-legal consequences.^213^, ^214^, ^215^, ^216^, ^217^ For example, a recent jury verdict of US$ 3 million was awarded for missed papilledema by an on-call neurology resident that caused permanent blindness (*Greer v. Serafini*, No. 2016007772, Mich. Cir. Ct. Wayne County 2016).

#### Further Development of ED Ocular Fundus Examination Protocols and Pathways

Given the fast-paced nature of ED workflows, seamless integration of ocular fundus screening into routine care is essential. Ideally, captured retinal images should be uploaded automatically directly to the EMR, enabling immediate access for ED physicians and remote interpretation by ophthalmologists. For this purpose, ocular fundus cameras equipped with DICOM capabilities are crucial for EMR compatibility, allowing on-call ophthalmologists to review images remotely. Furthermore, enabling ED providers to order ocular fundus imaging directly through the EMR is necessary to streamline workflow.^62^

There is now a growing body of studies investigating implementation pathways and protocols for ocular fundus examination. International guidelines have been developed regarding the indications and protocols for ocular fundus examinations in EDs.^218^^,^^219^ Of note, bedside direct fundoscopy was primarily recommended in the guidelines, likely due to the limited availability of retinal imaging devices in EDs. A few protocols have been proposed for ocular fundus cameras with and without OCT. For example, the teams from New York Eye and Ear Infirmary of Mount Sinai, Kaiser Permanente North California, and Emory University School of Medicine have introduced protocols for retinal artery occlusions, in which patients with acute painless vision loss are triaged to undergo emergent ocular fundus imaging in the ED.^110^, ^111^, ^112^ Similarly, a papilledema protocol using NMFP-OCT was instituted, significantly reducing ED length of stay and minimizing in-person consultations.^70^^,^^71^^,^^220^ These studies support the importance of ED access to ocular fundus imaging for prompt identification of ophthalmic and neurologic emergencies. Nonetheless, further evidence is required to refine screening protocols and care pathways for retinal imaging and to support their integration with advanced technologies such as teleophthalmology, automated systems, and AI, thereby enhancing clinical utility.

#### AI for Fundus Examination in the ED

**Figure 4:**
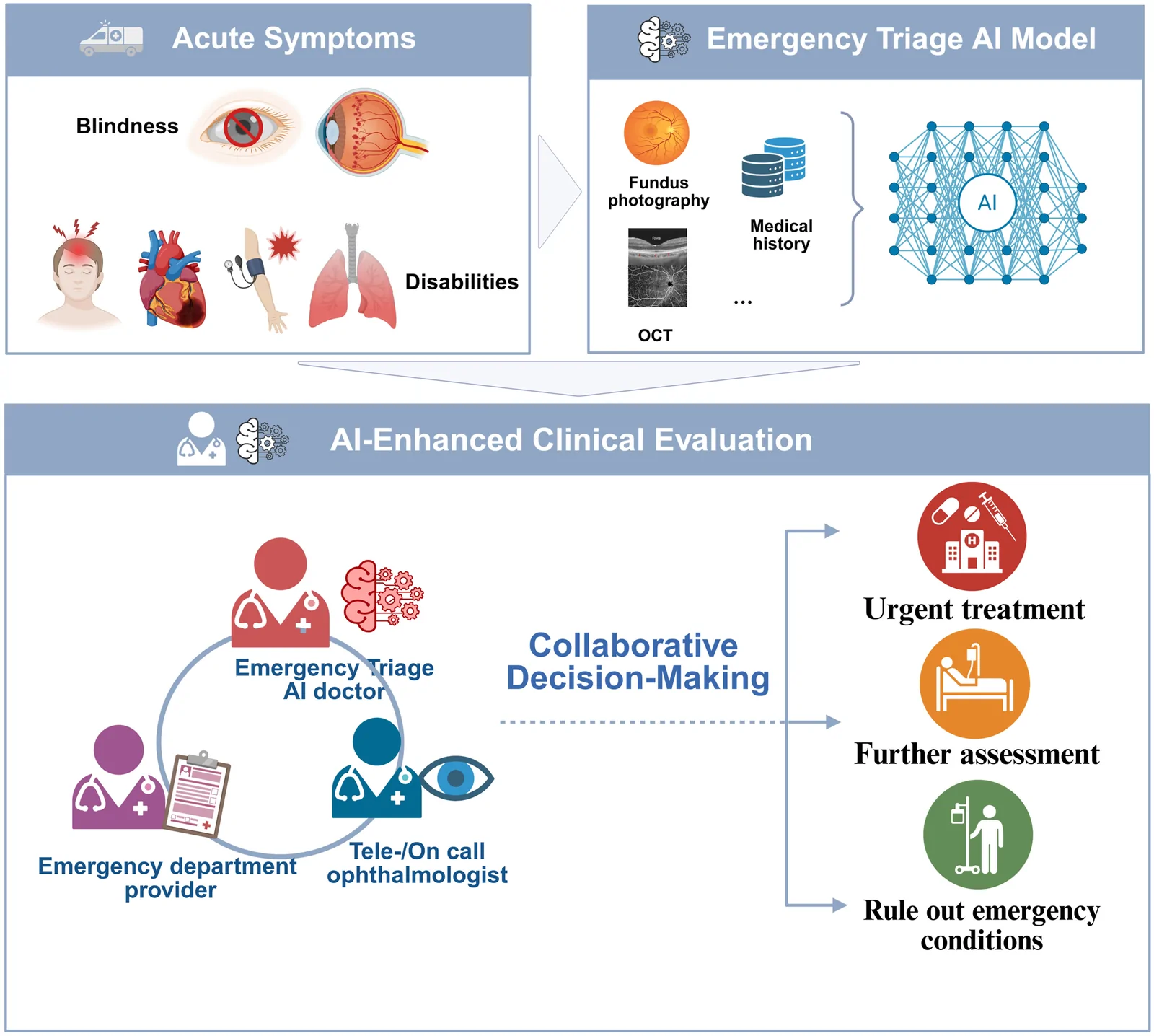
Proposed roadmap for implementation of ocular fundus imaging and AI in the ED. This figure was created with BioRender.com. AI = artificial intelligence; ED = emergency department.

The integration of AI with ocular imaging holds transformative potential for ED settings. Artificial intelligence–based vision models can aid in the detection of urgent conditions from ocular imaging, while LLMs can support clinical decision-making by assisting in diagnosis, triage, and management planning. For instance, Li et al demonstrated the successful integration of image-based DL and LLM within primary diabetes care, underscoring the potential for combining image-based DL and LLMs in the ED. This integrative system assists physicians by enhancing physician support in diagnostic reporting and follow-up care recommendations,^221^ thereby streamlining patient management in the busy ED. Figure 4 presents a proposed roadmap for integrating ocular fundus imaging and AI into the ED workflow. In patients with ocular, neurological, or other acute presentations, AI-assisted analysis of ocular fundus images, potentially augmented by multimodal data, can support ED providers and ophthalmologists by facilitating timely triage and collaborative decision-making, ultimately enhancing efficiency and patient outcomes in acute care.

Of note, even though there is great potential from AI and LLMs, their utility in EDs should be evaluated through clinical applicability implementation. Empirical evidence indicates that their accuracy could decline when prospectively deployed due to shifts in workflows, patient population differences, and varying data infrastructure between retrospective and real-time settings.^222^ Findings highlight that AI systems can suffer unexpected performance degradation when exposed to clinical applicability data over time, reinforcing the need for prospective validation and ongoing model monitoring.^223^

Finally, future efforts should focus on developing validated implementation pathways that enable real-time clinical use, support continuous performance monitoring, and ensure secure data handling, particularly in cloud-based and remote-access settings. Beyond technical readiness, successful integration of AI into emergency care requires collaboration across disciplines. Engaging domain experts and key stakeholders (e.g., emergency providers, ophthalmologists/optometrists, information technology professionals, and health administrators) is essential to gather context-specific insights and codevelop protocols suited to clinical applicability ED workflows.^224^ Such a multidisciplinary approach will help ensure that AI-assisted retinal examination delivers technically robust, clinically relevant, and patient-centered care.

### Conclusion

As multimodal AI and accessible retinal imaging continue to converge, the ED may become a key setting for redefining the clinical utility of ocular fundus assessment in acute care. Taken together, these developments suggest that AI systems capable of integrating imaging with broader clinical data and generating structured outputs may support more efficient interpretation and decision-making within ED workflows, while advances in user-friendly imaging may enable more consistent and scalable use in time-sensitive settings. However, substantial challenges remain, including clinical validation, workflow integration, and ethical, legal, and technical considerations, and careful evaluation will be essential before widespread implementation.
